# Accessibility crisis of essential medicines at Sudanese primary healthcare facilities: a cross-sectional drugs’ dispensaries assessment and patients’ perspectives

**DOI:** 10.1186/s12939-023-02009-y

**Published:** 2023-10-17

**Authors:** Lina Hemmeda, Abubaker E. A. Koko, Radia F. Mohamed, Yousra Ibrahim Abdallah Mohammed, Abeer Osman Mukhtar Elabid, Alaa T. Omer, Amna Abdel Rafea Al Hashemi Hamida, Aya M. Haiba, Eithar M. Ali, Istabraq I. Abdelgadir, Reem M. Al Fanob, Saja S. Mohamed Almahadi, Sara Ali, Suzan A. A Mahgoub

**Affiliations:** Faculty of Medicine, University of Khartoum, Khartoum, Sudan

**Keywords:** Essential medicines, Primary healthcare services, Affordability, Universal health coverage, Sudan’s healthcare

## Abstract

**Background:**

Access to essential medicines is a critical component of universal health coverage. However, the availability of essential medicines in Sudan isn’t well studied. As well, most Sudanese people lack health insurance, making out-of-pocket spending the primary source of drug financing. Therefore, the affordability of medicines in Sudan is questionable, with only 30% of the total population being covered by a public health service or public health insurance. We undertook this study to assess the availability and prices of essential medicines in public-sector health facilities in Khartoum state. Moreover, this study aims at assessing patients’ perceived affordability of essential medicines, and accommodation and acceptability of the public facility.

**Methods:**

A cross-sectional study was carried out at 30 primary healthcare facilities’ drug dispensaries across three districts in Khartoum state. Within each Centre’s dispensary unit, a standardized checklist evaluated the availability and affordability of 21 essential medicines selected from Sudan’s national essential medicines list and assessed their storage conditions. Furthermore, 630 patients were selected from all dispensary units for an exit interview that assessed their perceived accessibility, acceptability, accommodation, and affordability of essential medicines. Data were collected through the Kobo toolbox and analyzed using SPSS version 26.

**Results:**

Participants’ ratings of accessibility, affordability, accommodation, and acceptability were 3.7/5, 1.5/4, 5/6, and 5.4/6, respectively, with a 26.7% full access and weak correlation between some of the indices. The overall availability of adults and pediatric medicines was 36.8% 6.7%, respectively. Cost of a single course of treatment for 10 and 16 drugs out of the 19 drugs consumed exceeds the daily wage of insured and uninsured patients, with a median price ratio of 16.4 and 62.8, respectively. Moreover, the dispensary area conditions were found to be of good quality, yet the storerooms were not functioning in 40% of the outlets.

**Conclusion:**

Patients had limited access to their needed drugs due to high prices and physical unavailability, and primary healthcare capacities are not meeting the demands of citizens. The outcomes for the patients’ access variables (accessibility, accommodation, acceptance, and affordability) are comparable to those in countries with low incomes. Ensuring access to free medicines is likely to improve patients’ satisfaction with healthcare services and reduce private expenditure on medicines, which is a long-term, sustainable way towards universal health coverage in Sudan.

**Supplementary Information:**

The online version contains supplementary material available at 10.1186/s12939-023-02009-y.

## Background

The community’s perception of the quality of healthcare is most directly measured by the availability of pharmaceuticals, and when essential medications (EM) are exhausted, the load on healthcare facilities decreases by 50–75% [1]. The World Health Organization (WHO) defines essential medications as those that address the population’s top healthcare requirements. They are chosen with consideration for their significance to public health, proof of their efficacy and safety, and comparative cost-effectiveness [2]. A key component of primary healthcare (PHC), according to the Alma-Ata Declaration, is having access to necessary pharmaceuticals [3]. Additionally, according to the United Nations (UN), expanding public access to drugs is a step toward achieving universal health coverage and a sign of progress made in the “right to health” movement [4]. Multidisciplinary resources should be mobilized to ensure adequate coverage for essential medicines, as access to pharmaceuticals depends on several economic, political, and social issues and requires joint efforts from the commercial and public sectors [5]. Many African countries responded to these recommendations by establishing national policies to control drug availability through the formulation of national essential medicine lists (NEMLs), despite these efforts, a survey conducted by the WHO revealed that the median availability of EMs in African countries was 61.5% [6].

In Sudan, the National Medical Supply Fund (NMSF) indirectly maintains public sector procurement through local and global bids. The commodities (78% of the list of essential medications) are mostly provided to state hospitals and public institutions. Sudan is entirely reliant on imported medications [7], with local manufacturers accounting for barely 5% of essential medication output in Sudan [8]. There is evidence that restricted local production is the principal barrier to accessing medications in many countries, including Sudan [9]. Furthermore, studies and reports inspecting the availability of essential medicines in Sudan are scarce and outdated; the only governmental endeavour was the introduction of an ER-RHAD-based healthcare program in response to the Bamako Initiative (BI), which was proposed in 1987 at the Annual Meeting of Health Ministers in Bamako. The ER-RHAD program reported that common drugs were available at a rate of 43.9% [10]. In furtherance, a national study published in 2009 reported an 85% availability rate for essential medicines in the primary and governmental sectors, but with low-quality storage for these medications [8]. As well, the WHO reported in their last assessment of 2018 a 48.6% national availability in Sudan [11]. Worldwide, it’s known that many people cannot afford medicines because of their high prices [2]. Budgets for pharmaceuticals are high across the board, which is more obvious in developing nations where expenditure on medications makes up a sizeable fraction of overall healthcare costs [4, 5, 11–14], ranging between 20% and 60% [12], compared to 18% in developed nations [15]. Nevertheless, the Sudanese pharmaceutical market is relatively modest, with the total value of the pharmaceutical market (TPM) according to National Medicines and Poisons Board (NMPB) figures projected to be US $650 million, with the public sector accounting for $155 million (24.0%) and the private sector accounting for $495 million (76.0%) of that total value [7]. Moreover, the 1995-founded National Health Insurance Fund (NHIF) is in charge of ensuring that insurance-covered people across the nation have access to basic medical care. Because since 2014, just 37.3% of people had insurance, out-of-pocket expenses accounted for the majority of the purchases made by the remaining population [16]. Although there are no official statistics on access to drugs in Sudan, estimates for household spending on medications in Khartoum state show that they make up 58% of all household healthcare spending. This makes it crucial to check how affordable pharmaceuticals are in Sudan, particularly given that poverty is pervasive and is thought to be at a rate of more than 50% [17].

In addition, the concept of EMs is acknowledged as an invaluable tool for promoting health equity and lack of access to essential medicines can lead to avoidable deaths and diseases, particularly among underprivileged people, hence exacerbating existing health inequities. Measuring their availability might thus shed light on the overall degree of equality. Inaccessibility in public facilities, particularly in a nation like Sudan, with a 50% poverty rate, will increase private sectors utilization and lead to medication monopolies among specific socioeconomic groups.

All these prompted us to evaluate the price and availability of vital drugs as well as the quality aspects of each PHC dispensary pharmacy. Furthermore, our research looked into the many drivers and barriers to the usage of public dispensaries, including the accommodation and acceptability of each dispensary.

## Methods

### Study concept

The study achieved its stated objective in part by implementing Penchansky and Thomas’ concept of health service accessibility, which emphasized the importance of analyzing all five dimensions of access: availability, accessibility, accommodation, affordability, and acceptability (Additional File 1: Table S1) [18, 19]. Their perspectives were based on the pharmaceutical service models of North America and Western Europe, which were both widely used to assess access to medicines. To assess the availability of public PHC outlet pharmacies, the authors used the World Health Organization & Health Action Initiative (WHO/HAI) standardized methodology [19]. For the remaining dimensions, a questionnaire was created.

### Study design and settings

This is an observational, cross-sectional, facility-based study conducted in primary healthcare centres in Khartoum state, the capital of Sudan, located in its heart at the confluence of the Blue Nile and White Nile. It contains seven districts. There are a total of 432 primary healthcare facilities, from centres to units and dressing stations, providing preventive and curative health services for insured and uninsured patients. According to WHO Sudan, there are 1.5 primary healthcare facilities for every 10,000 people in Sudan. They are run by physicians and offer packages of services such as childhood immunization, nutrition, reproductive health (RH), integrated management of childhood immunization (IMCI), management of common illnesses, and prescription of necessary drugs [20].

Data were collected between October 11 and October 31, 2022. We followed the STROBE (Strengthening the Reporting of Observational Studies in Epidemiology) guidelines(Additional File 2).

### Participants

We included all patients aged 18 years and above who were dispensing medicines from the Centre’s outlet dispensary. We included the centres fulfilling the following: (a) governmental primary health care centres run by physicians in Khartoum State; (b) containing outlet pharmacies or medicine dispensaries within them; (c) being active within the past 6 months; and (d) having patients rate of more than 30 patients per day. According to specialists, centres with less than 30 patients per day tend to be irregularly open; only on specific days and hours; and their pharmacies are inoperable.

Patients who took part in the pilot study were omitted, as were those who were seriously unwell because collecting data from them was likely to be impossible. Additionally, all facilities classified as being below primary healthcare facilities in the level of care pyramid were omitted, including PHC units run by community health workers, dressing stations run by nurses, and dispensaries run by medical assistants.

### Essential medicines included

Fifteen key medicines (19 dosage formulae) were selected as per the WHO recommendations in their operational package for pharmaceutical situation assessment [21]. To ensure medications align with the acute, chronic, and endemic disease map of Sudan, an advisory group of four experts, including pharmacists, family physicians, and academics, was asked to independently highlight the 15 most needed medications to be included. Their selections were cross-matched by the researchers, and the final 15 medicines selected were listed. Of the 15 medicines, 10 were listed in the global medicine list of the WHO / HAI according to the disease spectrum and the necessity for basic medical care worldwide [19]. All of the medicines were listed in Sudan’s last (2019) NEML [22].

### Sample size and sampling technique

This study targeted primary healthcare centres and their adult attendants as sources of data. All primary healthcare centres in Khartoum were considered as the sampling frame, with individual healthcare centres being the sampling unit.

The estimated sample size of health centres was chosen with a 95% confidence interval and 15% margin of error (e), from 91 total eligible centres (N). The target sample size was therefore 30 centres derived from the following simple formula [23]:

1 $$n = \frac{N}{{1 + N{{\left( e \right)}^2}}} = 30$$

For the patients sample the following formula was used$, n =\frac{{z}^{2}P\left(1-P\right)}{{e}^{2}}$ with a 95% confidence interval (CI), 50% response distribution, and 5% margin of error; a sample of 384 was considered as the minimal sampling to represent the study population. by multiplying it by 1.2 design effect and considering a 35% non-response rate. the final sample size was 630.

Multi-stage cluster sampling was used to select the sample facilities and patients. Stage one was the random selection of three representative localities from the seven localities of Khartoum state. Omdurman, Khartoum North (Bahri), and Khartoum were selected using simple random sampling. Stage two was the selection of the PHC centres; using probability proportionate to size, 15 centres from Khartoum, seven from Omdurman, and eight from Bahri were randomly selected. Stage three was the selection of patients, due to the approximate equal patient rate at each centre, the sample size was divided relatively equally between the 30 centres. Using the relative patient rate at each outlet pharmacy, an interval was created by each data collector, and a systematic random sampling method was used. Ultimately, at least 13 responses were collected from each dispensary unit.

### Data collection tools and techniques

For the patients’ data, a structured and pre-tested questionnaire was adopted from the Brazilian PAUMA study “National Survey on Access, Use, and Promotion of Rational Use of Medicines “ [18], which was also dependent on Penshansky and Thomas’ concept. To verify the precision and reliability of the PAUMA instrument we used in this study, and to ensure its cultural suitability, an expert panel assessed and confirmed the instrument’s content. The questionnaire was then translated to Arabic by the study author and translated back to English by language experts; the two copies were compared for reliability. Pilot research was then carried out among a group of 50 patients from different primary healthcare centres. The questionnaire was then edited by the authors accordingly (Additional File 3: Patients’ Questionnaire). The Kobotoolbox application, an offline mobile data collection app for epidemiological surveys, was thoroughly explained to the collectors and subsequently used for patients’ data. Sociodemographic and a question about the insurance status of the respondent were added to the original PAUMA questionnaire, which included the following sections: accessibility; accommodation; affordability; and acceptability.

For the drugs dispensaries’ assessment, Following the adoption of the WHO/HAI standardized survey forms [19] and the finalization of the list of key essential medications, data collectors underwent extensive training sessions explaining the purpose of the study and discussing the WHO/HAI approach for pricing and availability measures [19]. Furthermore, in order to acquaint them with the method, the lead investigator, L.H., conducted a pilot collection, and the gathered data were then reviewed with the collectors. The key elements of the dispensary survey forms were: basic demographic information; availability of the chosen medications; costs of each medicine’s dosage form; and the appropriateness of the conservative conditions at the storerooms and dispensary units.

### Measures and analysis

*Patients’ data* was downloaded from KoboCollect into an Excel sheet file and then cleaned manually. Analysis was performed using Statistical Package for Social Sciences version 26 (SPSS 26). All missed variables were coded. Descriptive statistics were used for the *patient’s* characteristics, the availability of drugs, and the mean of the prices; the outcomes were displayed in tables and figures. Analysis of variance (ANOVA), and independent sample t-tests,were used to expore differences in indices scores according to different sociodemographic characteristics of the participants, while Spearman’s rho correlation tests were used to find the association between the different study variables. Scores for assessing accessibility, acceptability, affordability, and accommodation were calculated by rating participants’ responses to respective items in the questionnaire, and higher scores *indicated* a better measure of assessment. Two items in the questionnaire were used to evaluate affordability (financial adversity due to health expenditure, and due to medication expenditure, with a total score of 4), while three items were used to assess accessibility (perceived distance, ease of transportation, and presence of directions to the dispensing unit, with a total score of 5), acceptability (patients’ rating of service quality, treatment of staff members, and respect of privacy, with a total score of 6), and accommodation (comfort, wait times, and perceived suitability of opening hours, with a total score of 6). The internal consistency of subscales was assessed by calculating the Cronbach alpha statistic (0.68) using pilot data.

#### Availability index

Availability of essential drugs refers to, by the WHO/HAI, the proportion of the surveyed institutions that can provide a certain drug to the total number of survey institutions [19]. The mean availability of the selected medicines was calculated per the WHO/HAI recommendations. Furthermore, we calculated the availability of each medicine as the per cent availability of the total assessed medicines at the surveyed PHC facility and compared the availability for the different study districts.

#### Affordability Index

Is defined by the WHO/HAI, as the affordability of an essential medication during a specific course of treatment, the total medicine cost for the treatment of a condition with standard dosages of medicines divided by the minimum daily wage for non-technical staff in government departments [19]. Each essential drug price for the complete treatment course was collected in the Sudanese SDG, then divided by 311 SDGs, which is the daily lowest-paid unskilled government worker wage for Sudan as extracted from the World Salaries website [24]. If the total expense of drug treatment is less than the aforementioned minimum daily income criterion, the drug is assumed to be more affordable, and vice versa. For price evaluation, the *median price ratio (MPR*), which indicated the ratio of one medicine’s unit price to the international reference price (IRP) [19], was utilized. MPR’s particular calculating formula is as follows: MPR = median unit price of the target drug within the survey range/international reference price × 100%. When comparing drug purchase price levels using MPR values, MPR = 1 is typically used as the threshold value. When this value is less than one, it means that the investigated drug price is lower than the international average standard, and vice versa. The WHO recommends that retail pricing of medications should not allow for an MPR in excess of 2 [25].

## Results

### Demographics and general information

The study assessed services at 30 primary healthcare centres distributed in Khartoum (52.5%), Khartoum North (28.1%), and Omdurman (19.4%) localities. The total number of respondents was 630, with a mean age of 43.7 ± 15 years, with more than two-thirds (69.7%) being females. Nearly 60% were unemployed, while 25.1% reported having no access to health insurance. 67.5% of the participants reported the purpose of their visit to be the attainment of a drug for a current (acute) illness (Table 1).

**Table 1:** Demographics and General Information

		N	N %	Mean (SD)
Locality	Omdurman	122	19.4%	
	Khartoum North	177	28.1%	
	Khartoum	331	52.5%	
Gender	Female	439	69.7%	
	Male	191	30.3%	
Age				43.7 (15)
Age group	<= 25	93	14.8%	
	26–44	229	36.3%	
	45–63	240	38.1%	
	64+	68	10.8%	
Marital status	Single	130	20.6%	
	Married	439	69.7%	
	Divorced	25	4.0%	
	Widowed	36	5.7%	
Educational level	Illiterate	78	12.4%	
	Primary	109	17.3%	
	Secondary	197	31.3%	
	University	205	32.5%	
	Higher Education	29	4.6%	
	Informal education ‘Khalwa’	12	1.9%	
Occupational status	Non-occupied	377	59.8%	
	Occupied	253	40.2%	
Do you have health insurance?	Yes	365	74.9%	
	No	122	25.1%	
How much does your household spend monthly on regular expenses in SDGs?				207,198.4 (154,136.6)
What is the purpose of your dispensary visit?	Dispensing regular medications	207	32.9%	
	Dispensing a currently prescribed (acute illness) medicine	425	67.5%	
	Dispensing over the counter medications	26	4.1%	

### Perceived accessibility, affordability, accommodation, and acceptability

Participants reported a mean accessibility score of 3.7 ± 1.0 out of 5.0, this is despite nearly a quarter of the participants (24.6%) reporting no access to their needed medications. More than half (51.3%) of the respondents reported that the PHC is far from their home, with 49.8% reaching the PHC by walking, and 75.1% having difficulty finding means of transportation to the PHC. Nearly three-quarters (72.4%) of participants reported the availability of signs in the PHC guiding to the dispensing unit (Table 2).

The mean affordability score was 1.5 ± 1.1 out of 4.0. Only 37.3% stated that health issues expenditure could sometimes interfere with other needs, and 79.6% of them attributed this to medications. Upon asking about drugs’ perceived expensiveness, only 1.3% reported receiving their medications free of charge, 48.3% on the other hand perceived their medicines as being expensive (Table 2).

**Table 2:** Results of Patients’ Accessibility and Affordability Dimensions

		N	N %	Mean (SD)
How do you perceive your access to your needed medications?	Full access	168	26.7%	
	Partial access	307	48.7%	
	No access	155	24.6%	
Is the primary healthcare center far from the patients’ house?	No	203	32.2%	
	More or less	104	16.5%	
	Yes	323	51.3%	
What is your mean of transport to reach the PHC center?	General	184	29.2%	
	Walking	314	49.8%	
	Private	90	14.3%	
	Others	42	6.7%	
Is it easy to find a transportation facility to go to the PHC center?	No	27	14.6%	
	More or less	19	10.3%	
	Yes	139	75.1%	
Are there any existing signs in the PHC center to find the medicines dispensing unit?	No	152	27.6%	
	Yes	399	72.4%	
Overall accessibility score				3.7 (1.0)
Were you ever not able to buy something important to cover expenses for any health problem?	Yes	235	37.3%	
	No	395	62.7%	
If yes, Were the medicines the problem that caused this expense	Yes	187	79.6%	
	No	48	20.4%	
Do you regard the medicines you buy as expensive	Yes	304	48.3%	
	More or less	99	15.7%	
	No	219	34.8%	
	I got it for free	8	1.3%	
Overall affordability score				1.5 (1.1)

Regarding Accommodation, the mean score was 5 ± 1.4 out of 6, with 74.4%, 84.1%, and 74.1% rating the dispensing unit as comfortable, clean, and having suitable opening hours, respectively. The mean waiting time for receiving medications was 21.1 ± 55.6 min, and only 13.5% of patients perceived the waiting time as long. Participants recorded a mean acceptability score of 5.4 ± 1.0 out of 6.0, with 91.4% stating that they were treated with courtesy, 74.9% rating the services as having adequate quality, and 89.4% thought that their privacy is conserved throughout the visit (Table 3).

**Table 3:** Results of Patients’ Accommodation and Acceptability Dimensions

		N	N %	Mean (SD)
Do you regard this dispensing unit as comfortable?	No	79	12.5%	
	More or less	82	13.0%	
	Yes	469	74.4%	
Do you regard this dispensing unit as clean?	No	36	5.7%	
	More or less	64	10.2%	
	Yes	530	84.1%	
Please estimate the waiting time from reaching the dispensing unit till receiving your medicines?				21.1 (55.6)
Do you perceive this time as longtime?	Yes	85	13.5%	
	More or less	60	9.5%	
	No	485	77.0%	
Are the opening hours of this dispensing unit suitable?	No	90	14.3%	
	More or less	73	11.6%	
	Yes	467	74.1%	
Overall Accommodation score				5.0 (1.4)
Do the staff of the dispensing unit treat patients with respect and courtesy?	No, never	14	2.2%	
	Yes, sometimes	39	6.2%	
	Yes, always	577	91.6%	
Do you regard the service presented at the PHC unit of good quality?	No	30	4.8%	
	More or less	128	20.3%	
	Yes	472	74.9%	
Is your privacy respected throughout the services?	No, never	41	6.5%	
	Yes, sometimes	24	3.8%	
	Yes, always	565	89.7%	
Overall acceptability score				5.4 (1.0)

A significant difference in accessibility, affordability, and acceptability scores was noted according to marital status (p = 0.047, p = 0.003, and p = 0.002), while affordability (p < 0.001), acceptability (p = 0.001), and accommodation (p < 0.001) scores were also different between localities (Tables 4 and 5). Moreover, the accommodation score was weakly positively correlated with the affordability (p < 0.001, rho = 0.204) and the acceptability (p < 0.001, rho = 0.35) scores, while there was a weak significant correlation between acceptability and affordability (p = 0.011, rho = 0.101) scores. (Additional File 1: Table S2).

**Table 4:** Factors Affecting Patients’ Accessibility and Affordability Scores

		Accessibility		Affordability	
		Mean (SD)	*p* (t/F)	Mean (SD)	P* (t/F)
Gender	Female	3.7 (0.95)	0.413 (-0.82)	1.56 (1.15)	0.167 (1.38)
	Male	3.84 (1.18)		1.42 (1.03)	
Marital status	Single	3.46 (1.0)	0.047* (2.69)	1.78 (1.04)	0.003* (4.75)
	Married	3.78 (1.0)		1.44 (1.1)	
	Divorced	3.50 (1.05)		1.88 (1.27)	
	Widowed	4.4 (0.97)		1.22 (1.24)	
Educational level	Illiterate	4.15 (0.99)	0.355 (1.114)	1.27 (1.14)	0.375 (1.071)
	Primary	3.81 (1.01)		1.55 (1.12)	
	Secondary	3.68 (1.01)		1.50 (1.10)	
	University	3.61 (1.04)		1.60 (1.11)	
	Higher Education	3.43 (0.79)		1.55 (1.24)	
	Informal education ‘Khalwa’	4.0 (1.0)		1.67 (0.98)	
Occupational status	Unemployed	3.75 (0.94)	0.813 (0.236)	1.52 (1.13)	0.889 (0.139)
	Employed	3.71 (1.1)		1.51 (1.0)	
Locality	Omdurman	3.85 (1.06)	0.236 (1.479)	1.48 (1.1)	< 0.001* (23.66)
	Khartoum North	3.91 (1.14)		1.98 (1.07)	
	Khartoum	3.63 (0.93)		1.29 (1.08)	
Do you have health insurance?	Yes	3.71 (1.03)	0.433 (-0.786)	1.53 (1.12)	0.703 (0.382)
	No	3.87 (0.88)		1.48 (1.16)	

*As for the tests, we used t-test when studying the difference in an index when the variable has two groups, and ANOVA when the variable has more than two

**Table 5:** Factors Affecting Patients’ Acceptability and Accommodation Scores

		Acceptability		Accommodation	
		Mean (SD)	*p* (t/F)	Mean (SD)	p* (t/F)
Gender	Female	5.4 (1.0)	0.586 (-0.544)	5.00 (1.44)	0.966 (-0.043)
	Male	5.5 (1.0)		5.01 (1.34)	
Marital status	Single	5.2 (1.2)	0.002* (4.96)	4.95 (1.34)	0.745 (0.41)
	Married	5.5 (1.0)		5.00 (1.43)	
	Divorced	5.6 (0.8)		5.28 (1.4)	
	Widowed	5.8 (0.6)		5.06 (1.47)	
Educational level		5.6 (0.8)	0.242 (1.348)	5.54 (0.91)	0.007* (3.234)
	Primary	5.6 (0.9)		5.08 (1.26)	
	Secondary	5.4 (1.1)		4.90 (1.49)	
	University	5.3 (1.1)		4.83 (1.54)	
	Higher Ecducation	5.4 (0.9)		5.07 (1.39)	
	Informal education ‘Khalwa’	5.7 (0.8)		5.17 (1.03)	
Occupational status	Non-occupied	5.4 (1.1)	0.269 (-1.107)	4.96 (1.45)	0.338 (-0.958)
	Occupied	5.5 (0.9)		5.07 (1.35)	
Locality	Omdurman	5.2 (1.3)	0.001* (7.09)	5.04 (1.28)	< 0.001* (14.8)
	Khartoum North	5.6 (0.8)		5.45 (0.99)	
	Khartoum	5.4 (1.0)		4.75 (1.58)	
Do you have health insurance?	Yes	5.4 (1.0)	0.378 (0.883)	4.93 (1.33)	0.372 (-0.894)
	No	5.3 (1.1)		5.07 (1.32)	

*As for the tests, we used t-test when studying the difference in an index when the variable has two groups, and ANOVA when the variable has more than two

### Drugs availability

A total of 15 essential drugs were assessed, some with two different forms making a total of 19 drugs. In addition, the availability of two pediatric drugs was evaluated. Overall availability of 36.8% was found for the 19 drugs, with 80.0% for folic acid and Artemether Lumefantrine tabs, 90.0% metronidazole, and 36.7% paracetamol tabs (Table 6). Moreover, Khartoum North district PHC dispensaries showed the highest total availability of 42.1% in comparison to Omdurman (36.84%) and Khartoum (34.03%) areas. For pediatric drugs, 0% availability was found for vitamin A caps, while 10% was the availability for both isoniazid and Co-trimoxazole suspensions (Additional File 1: Table S3). In addition, medication availability was reassessed after grouping the medications into categories, antimalarial displayed the highest availability (48.3%), followed by antibiotics (39.5%) (Additional File 1: Table S4).

### Prices as equivalent to the daily wage

Comparing the price of medicine, both insured and uninsured costs, required for a single course of treatment with the minimum wage of an unskilled governmental worker, it was found that the total cost of a single course of treatment for 10 and 16 drugs out of the 19 drugs consumed exceeds the daily wage of insured and uninsured patients respectively. The most unaffordable drug was acetylsalicylic acid tabs with 30 days of treatment being 15.8 times the daily wage of an insured patient, and 57.20 times that of an uninsured patient. Benzyl benzoate lotion, Lidocaine injection, and Mebendazole suspension on the other hand were the most affordable of the list, with the first two being free of charge, and the last consuming 0.19 and 0.76 of the daily wages of insured and uninsured patients, respectively. More details are shown in (Table 6) (Additional File 1: Table S5).

**Fig. 1:**
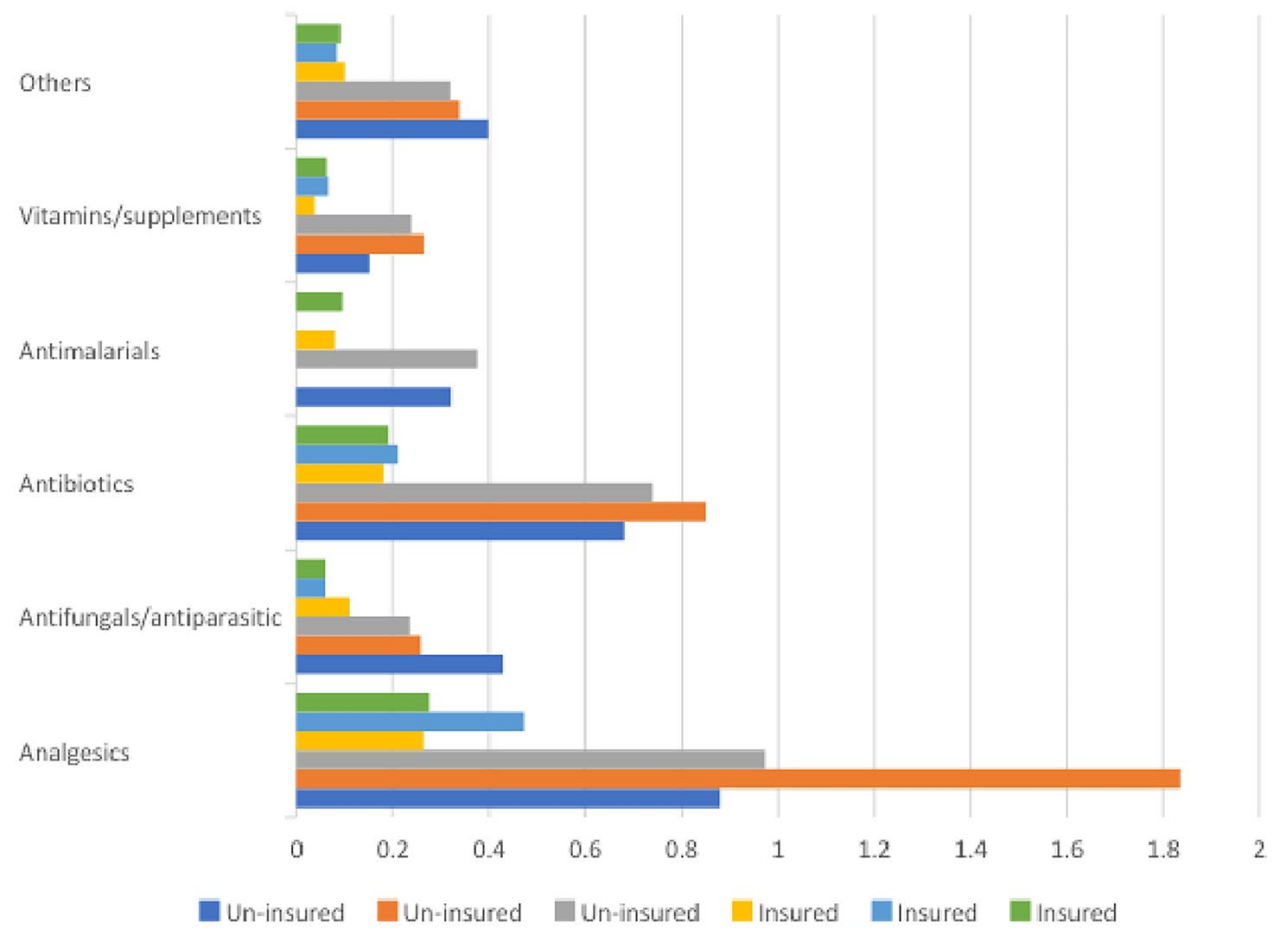
Prices of drug categories (in days) as equivalent to the lowest daily salary

Furthermore, through displaying drugs by their disease categories, analgesics appear to consume the highest by the uninsured patients who purchase from Khartoum North district dispensaries (12.81 days) and the lowest by insured patients purchasing from Khartoum dispensaries (1.69 days). Overall, antifungal (12.9 days) and analgesics (12.81 days) were the most unaffordable drug categories. (Fig. 1) shows more details on the prices of each drugs categories.

### The median price ratio (MRP)

The mean MRP for the 19 medications is 16.4 with insurance and 62.81 without. That is, the average price for essential medications is 16.4 times higher than the international reference price for these drugs. Four of the 19 medications (Lidocaine injection (0), Artesunate injection, Artemether Lumefantrine tablets, and Benzyl benzoate lotion) had MRPs of less than two for both insured and uninsured prices. Ringer lactate came out to be 125.49 times its international.

reference price when purchased with insurance coverage; MRP = 125.49. In the context of pediatric medications, MRPs of 0.05 and 0.21 showed up for insured and uninsured pricing, respectively, equating to 5% and 21% of the international reference price. (Table 6).

**Table 6:** Essential Medicines (EMs) Availability Percentages, Prices Relative to Daily Wages, and Median Price Ratio (MRP) for Both Insured and Uninsured Costs

		Insured		Uninsured	
	Availability (%) N = 30	Mean in days (S. D)	MPR	Mean in days (S. D)	MRP
Paracetamol tabs	11 (36.7%)	1.05 (0.04)	18.25	3.87 (0.36)	68.83
Mebendazole suspension	9 (30.0%)	0.19 (0.18)	0.56	0.76 (0.58)	1.97
Mebendazole tabs	10 (33.3%)	0.33 (0.21)	3.19	1.14 (0.60)	11.16
acetyl salicylic acid tabs	9 (30.0%)	15.8 (5.88)	89.10	57.20 (30.06)	325.81
Amoxicillin tabs/caps	26 (86.7%)	3.92 (2.82)	10.20	15.26 (10.79)	39.72
Amoxicillin suspension	13 (43.3%)	0.79 (0.50)	13.50	3.36 (1.80)	57.20
Lidocaine injection	2 (6.7%)	0 (0.00)	0	0 (0.00)	0
Metronidazole tabs	27 (90.0%)	1.24 (0.18)	8.19	5.23 (0.32)	34.61
Artesunate injection	5 (16.7%)	0.54 (0.56)	0.00	2.10 (2.25)	0.011
Co-trimoxazole suspension	4 (13.3%)	0.35 (0.25)	2.28	1.35 (0.90)	8.80
Artemether Lumefantrine tabs	24 (80.0%)	0.28 (0.48)	0.00	1.17 (2.02)	0.02
Ferrous salt tabs	5 (16.7%)	2.1 (2.26)	12.73	8 (8.60)	48.50
Ferrous salt oral solution	6 (20.0%)	1.9 (3.03)	2.77	7.60 (12.14)	11.08
Folic acid tabs	24 (80.0%)	2.6 (0.17)	14.81	10.40 (0.62)	59.24
Benzyl benzoate lotion	2 (6.7%)	0 (0.00)	0	0 (0.00)	0
Fusidic acid ointment	2 (6.7%)	0.91 (0.92)	0.85	3.76 (3.70)	3.49
Salbutamol tabs	6 (20.0%)	1.4 (1.21)	10.22	5.30 (4.66)	38.69
Ciprofloxacin eye drops	7 (23.3%)	1.03 (0.90)	1.11	3.92 (3.47)	4.25
Ringer lactate infusion	18 (60.0%)	1.61 (0.12)	125.49	6.16 (0.55)	480.14
*Total Availability (%)*	*210 (36.8%)*				
Co-trimoxazole suspension for ped	3 (10.0%)	1.28 (1.20)	0.09	2.92 (2.56)	0.19
Isoniazid tabs	3 (10.0%)	3.8 (6.58)	0.00	21.5 (22.74)	0.03
*Pediatric Availability*	*6 (10%)*				

### Adequacy of dispensary units conservative conditions

Each outlet pharmacy’s storeroom and dispensary were evaluated for conservative conditions. Only 40% [12] of the outlet pharmacies have a functioning storeroom, with 91.7% having a temperature control method, while temperature charts were found only in 8.3% (Additional File 1: Table S6). In regards to the dispensary area, all 30 units had a temperature control method (100%), 90% of them had windows or air vents, and 70% did not expose drugs to direct sunlight. On the other hand, only 33.3% had temperature charts, and 70% had their staff handle the tablets by hand (Additional File 1: Table S7).

## Discussion

This study has evaluated patients’ accessibility to essential medicines at the primary healthcare level in Khartoum, Sudan, through a full analysis of the availability, acceptability, accommodation, and affordability. And to make relevant suggestions to improve the current situation. We have attempted to reflect the exact situation of availability and prices of 19 EMs in 30 outlet dispensaries of Sudan PHCs. To the best of our knowledge, this is the first study that evaluates accessibility by both examining patients’ perspectives and analyzing pharmacy circumstances in Sudan and maybe in the African region.

According to a Tanzanian study, there is approximately a three-quarter drop-in rate in health facilities when essential drugs are used up [1], so EMs are regarded as the most visible indicator of healthcare quality as perceived by the community. The overall availability of public primary healthcare dispensaries in Khartoum state was found to be 36.8%, which is markedly below the WHO standards of 80% [8]. A study performed in 2009 based on the same methodology reported 79.14% availability at Khartoum state public facilities [8], which is well above the current rate. This remarkable difference may be due to the deteriorated economic status of Sudan after the separation of South Sudan and the loss of more than half of the oil revenue [26]. This deterioration is further confirmed by another study conducted in 2013, which reported a 68.2% availability of EMs in Khartoum State [27]. The WHO reported in their last assessment of 2018 a 48.6% national availability in Sudan [11]. Another explanation could be the low local supply of drugs in Sudan, currently at 5% [8]. There is evidence that limited local manufacturing is the main hurdle to accessing medicines not only in Sudan but in many other countries [9]. A third proposed explanation may be that the centralized procurement policy of the Central Medicine Supply Public Corporation (CMSPC) harms PHC pharmacies and hinders the purchase of medicines [28]. This centralized procurement was also apparent in the availability trends in the three included districts, with all having almost the same availability results. The 36.8% availability of the current study is similar to a Ugandan study, which reported availability of 40% [9], yet contradicts a Tanzanian study of more than 70% availability in their facilities [1]. Similar findings were also reported by other low- or low-middle-income countries; a study in India documented a slightly higher availability of 45.2% [29], and a Bangladesh study reported that almost 85% of their urban clinics had availability of less than 75% without specifying the exact level of availability [30]. Moreover, a study in Brazil, an upper middle-income country, found 61% of drugs were available at their PHCs. They reported financial insufficiency as the primary hindrance to drug availability at their PHCs [31]. This, however, isn’t the case in Sudan, as all 30 of the included PHC centres stated that they receive the drugs for free from a Federal Ministry of Health-affiliated body. In comparison to Middle Eastern countries, Sudan’s availability is among the lowest, being higher just than Libya (13%), and lower than Iran (96.7%), Palestine (92%), KSA, and UAE (100%) [11] [24], which is expected as most of these countries are upper-middle income countries. By looking at a high-income country example, China’s drug availability has fluctuated throughout the years, with many essential medicines falling between 2010 and 2012 at primary hospitals, from 27 to 23% for the cheapest generics. The median availability of generic medications then grew in 2018 until it steadied at about 55% in 2021 [31–34]. This unsteady nature of drug availability could be due to the variation in countries’ economies and inflations, which could positively or negatively affect the pharmaceutical market. In addition, a noticeably low availability was noticed for analgesics and antibiotics, which were only available in a third of the included public dispensaries. This could be due to the high consumption rate of these drug categories, so they tend to quickly run out of stock. Regarding pediatric medicines, a very low level of availability was detected, with a 10% total rate; this could be due to the use of WHO-standardized pediatric medicines in the assessment, as Sudan’s NEML doesn’t specify pediatric drugs, which could be inconsistent with the pediatric disease map of Sudan.

In terms of prices, most of the medicines were found to be unaffordable, with 10 and 16 EM insured and uninsured costs respectively above the lowest-paid daily wage. In particular, acetylsalicylic acid and amoxicillin treatment courses appeared to be the most expensive. The treatment course for adult pneumonia costs almost 4 days of salary, which is consistent with the uniformly unaffordable treatment of pneumonia in the reviewed literature [27, 29–35]. Unfortunately, most of the Sudanese people earn less than the lowest-paid governmental salary, and with 47% of the population living below the poverty line, these unaffordable prices could be catastrophic [17]. This claim is further reimbursed by the fact that more than three-quarters of those having difficulty paying for their medical expenses in this study sample claimed that unaffordable medications were the primary obstacle.

The affordability crisis seems to be a deep and rooted issue in Sudan; in 2007, an assembly held by the WHO regional office for the East Mediterranean on medicine prices and access to medicines in the region announced Sudan’s medicine prices to be the highest in the region [36]. In response to that, the government amended and updated an already established price regulatory act, the Medicines and Poisons Act [37]. Also, there is a 35% markup by the CMSPC on medicines before passing them to Khartoum Federal state, which adds on another 35% markup before selling them to public facilities, and the price of medicines increases by 2 to 3 folds before reaching the end users [38]. Showing a clear inability of the National Medicines and Poisons Board (NMPB), the drug regulatory agency of Sudan, to control the prices of imported medicines [39]. This assertion is further supported by the finding of this study that Sudan’s drug prices are 16.4 times their international counterparts for insured prices (MRP = 16.4), this MRP is eight folds higher than the one reported by Ismaeil and Mousnad in their 2014 assessment of prices in the public facilities of Khartoum state [40].

Another important finding of this study was the evaluation of the sufficiency of storage conditions for all of the investigated pharmaceuticals, which found high positive conservative conditions, with most of the dispensary rooms being well equipped to retain the drugs in their right forms. On the other hand, we observed a major flaw in the storage conditions, with most of the storerooms being unprepared to keep the medications. According to the pharmacists, this is primarily due to the storerooms not being under use, as the dispensaries receive a modest supply of drugs that only fill the dispensary area and the storerooms aren’t currently under service. Another study conducted in 2009 had the same conclusion [8], which may point to the rooted problem of the dispensaries’ storerooms.

Besides that, to discover elements and circumstances that go beyond the straightforward provision of drugs, a multidimensional analysis of access to medicines from the perspectives of the patients is crucial. Patients’ overall perceived accessibility was low, with only one quarter stating that they have full access to medicines, a finding that is in alliance with the low availability. However, most other low- or low-middle-income countries’ studies reported relatively higher patients satisfaction in terms of medicine availability [9, 18]. For high-income countries, low perceived availability was reported among Finnish [41] PHC dwellings in contrast to high satisfaction among Chinese [33]. Additionally, about half of the study sample walks to the PHC, indicating suitable territorial access to the centre; this is confirmed further by half of them admitting that the centre isn’t far away from their houses; similar geographical access was also reported by two other studies [18, 42]. Nevertheless, patients’ perspectives about medications could give insight into their availability at public dispensaries; a longitudinal Chinese study documented a tendency for people to pick up their medicines from private pharmacies despite high availability in public ones [33]. This finding could indicate that avoidance of lower levels of care is a culture regardless of the nation’s development.

Based on our findings, state governments should evaluate their procurement systems to ensure efficiencies and make necessary reforms to improve availability. Price regulations for essential medicines should be strengthened, and a dynamic, open, and transparent monitoring system for prices is needed to guarantee access to affordable essential medicine. We also highly recommend that the health insurance scheme be further expanded to be in line with the high poverty rates in the country. At the PHC level, primary healthcare dispensaries’ infrastructure should be reassessed and repaired to ensure adequate storage conditions. We believe that the results of such analyses can guide operational research and inform decision-making, investment, and priority-setting.

Finally, this study was limited to the public dispensaries of Khartoum state, which supplies the bulk of Sudan’s health services, and contains 25% of PHC facilities [43], due to the unfortunate centralized distribution. On the other hand, rural regions are home to 66.8% of the Sudanese population [44]. As a result, the lack of available medication and high prescription prices in the centralized region may provide a clue about Sudan’s overall tragedy, and it inadvertently demonstrates the significant inequities that rural populations endure. Furthermore, The WHO/HAI methodology is based on on-shelf availability; therefore, this study might not indicate stock availability; however, this methodology is widely used in the literature, and it puts our findings in a global context.

## Conclusion

This report indicated that patients at public health facilities had limited access to their needed drugs due to high prices and widespread poverty. The affordability of medications for some drug categories, such as antimalarials, is adequate, while for several others, such as analgesics and antibiotics, is higher than their international reference and still requires improvement. Overall, it appears that the population’s needs are not being met by PHC capabilities. A long-term, sustainable method to lower private healthcare spending is to strengthen the public sector’s access to medications, so the need for more funding for medical care cannot be overstated. For patients’ survey, the outcomes for the access factors (accessibility, accommodation, acceptability, and affordability) are essentially similar to those in developing nations. However, the scarcity of essential medications at public health facilities continues to seriously impede access to medications, indicating that Sudan’s PHCs continue to face difficulties in this area.

### Electronic supplementary material

Below is the link to the electronic supplementary material.

Supplementary Material 1

Supplementary Material 2

Supplementary Material 3

## Data Availability

The data that supports the findings of this study are available from the corresponding author upon reasonable request.

## Abbreviations

EMs: Essential Medicines
WHO: World Health Organization
PHC: Primary Health Care
UN: United Nations
NEMLs: National Essential Medicines Lists
NMSF: National Medical Supply Fund
BI: Bamako Initiative
TPM: Total Value of Pharmaceutical Market
NHIF: National Health Insurance Fund
HAI: Health Action Initiative
RH: Reproductive Health
IMCI: Integrated Management of Childhood Immunization
