# Supplementary material for: Accessibility crisis of essential medicines at Sudanese primary healthcare facilities: a cross-sectional drugs’ dispensaries assessment and patients’ perspectives
Source: Int J Equity Health. 2023 Oct 17;22:216. doi: 10.1186/s12939-023-02009-y (PMC10583350; doi:10.1186/s12939-023-02009-y)
Supplement: Supplementary file 1 — Supplementary Material 1 [file 12939_2023_2009_MOESM1_ESM.docx]

**Supplementary Tables:**

***Table S1: Definitions of the Four Study Dimensions***

| **Dimension** | **Definition** |
| --- | --- |
| Availability | Relationship between the type of services and volume of existing resources according to the needs and volume of patients. |
| Accessibility | Relationship between the location of the service and location of patients, considering resources of users of transportation, travel time, distance, and cost. |
| Accommodation | Represents the relationship between the way the services are organized to receive patients and the ability of patients to adapt to this organization. |
| Affordability | Relationship between the cost of services and payment capacity of the patient or client. |
| Acceptability | Represents the attitudes of individuals and providers regarding the characteristics and practices of each one. |

***Table S2: Correlational Statistics between the Study Dimensions***

|  | | | **Accessibility** | **Acceptability** | **Affordability** | **Accommodation** |
| --- | --- | --- | --- | --- | --- | --- |
| **Spearman's rho** | **Accessibility** | **Coefficient** | 1.000 | .104 | .102 | .029 |
|  |  | **Sig. (2-tailed)** | . | .157 | .167 | .698 |
|  | **Acceptability** | **Coefficient** | .104 | 1.000 | .101^*^ | .350^**^ |
|  |  | **Sig. (2-tailed)** | .157 | . | .011 | .000 |
|  | **Affordability** | **Coefficient** | .102 | .101^*^ | 1.000 | .204^**^ |
|  |  | **Sig. (2-tailed)** | .167 | .011 | . | .000 |
|  | **Accommodation** | **Coefficient** | .029 | .350^**^ | .204^**^ | 1.000 |
|  |  | **Sig. (2-tailed)** | .698 | .000 | .000 | . |
| *. Correlation is significant at the 0.05 level (2-tailed). | | | | | | |
| **. Correlation is significant at the 0.01 level (2-tailed). | | | | | | |

***Table S3: Essential Medicines (EMs) Availability in the Three Included Localities***

|  | |  |  |  |
| --- | --- | --- | --- | --- |
|  |  | **Omdurman n=7** | **Khartoum North n=8** | **Khartoum n=15** |
|  |  |  |  |  |
| **Drug availability** | **Paracetamol tabs** | 2 (28.6%) | 3 (37.5%) | 6 (40.0%) |
|  | **Mebendazole suspension** | 3 (42.9%) | 3 (37.5%) | 3 (20.0%) |
|  | **Mebendazole tabs** | 3 (42.9%) | 2 (25.0%) | 5 (33.3%) |
|  | **acetyl salicylic acid tabs** | 3 (42.9%) | 3 (37.5%) | 3 (20.0%) |
|  | **Amoxicillin tabs/caps** | 6 (85.7%) | 8 (100%) | 12 (80.0%) |
|  | **Amoxicillin suspension** | 3 (42.9%) | 5 (62.5%) | 5 (33.3%) |
|  | **Lidocaine injection** | 0 (0.0%) | 2 (25.0%) | 0 (0.0%) |
|  | **Metronidazole tabs** | 7 (100%) | 7 (87.5%) | 13 (86.7%) |
|  | **Artesunate injection** | 2 (28.6%) | 1 (12.5%) | 2 (13.3%) |
|  | **Co-trimoxazole suspension** | 1 (14.3) | 1 (12.5%) | 2 (13.3%) |
|  | **Artemether Lumefantrine tabs** | 6 (85.7%) | 7 (87.5%) | 11 (73.3%) |
|  | **Ferrous salt tabs** | 1 (14.3%) | 1 (12.5%) | 3 (20.0%) |
|  | **Ferrous salt oral solution** | 1 (14.3%) | 3 (37.5%) | 2 (13.3%) |
|  | **Folic acid tabs** | 5 (71.4%) | 7 (87.5%) | 12 (80.0%) |
|  | **Benzyl benzoate lotion** | 1 (14.3%) | 0 (0.0%) | 1 (6.7%) |
|  | **Fusidic acid ointment** | 0 (0.0%) | 1 (12.5%) | 1 (6.7%) |
|  | **Salbutamol tabs** | 1 (14.3%) | 1 (12.5%) | 4 (40.0%) |
|  | **Ciprofloxacin eye drops** | 0 (0.0%) | 4 (50.0%) | 3 (20.0%) |
|  | **Ringer lactate infusion** | 4 (57.1%) | 5 (62.5%) | 9 (60.0%) |
|  | ***Total availability*** | ***49 (36.8)*** | ***64 (42.1%)*** | ***97 (34.0%)*** |
|  | **Co-trimoxazole suspension for ped** | 1 (14.3%) | 0 (0.0%) | 2 (13.3%) |
|  | **Isoniazid tabs** | 1 (14.3%) | 0 (0.0%) | 2 (13.3%) |
|  | ***Pediatric availability*** | ***2 (14.3%)*** | ***0 (0.0%)*** | ***4 (13.3%)*** |

***Table S4: Availability Displayed by Categories***

|  | | **Locality** | | | |
| --- | --- | --- | --- | --- | --- |
|  |  | **Omdurman** | **Khartoum North** | **Khartoum** | **Total** |
|  |  | **Availability N (%)** | | | |
| **Drug availability** | **Analgesics** | 5 (35.7%) | 6 (37.5%) | 9 (30.0%) | 20 (33.3%) |
|  | **Antifungals/antiparasitic** | 14 (40.0%) | 13 (32.5%) | 23 (30.7%) | 50 (34.4%) |
|  | **Antibiotics** | 19 (38.8%) | 25 (44.6%) | 39 (37.1%) | 83 (39.5%) |
|  | **Antimalarials** | 8 (57.1%) | 8 (50.0%) | 13 (43.3%) | 29 (48.3%) |
|  | **Vitamins/supplements** | 7 (25.0%) | 11 (34.4%) | 17 (28.3%) | 35 (29.2%) |
|  | **Others** | 5 (23.8%) | 8 (33.3%) | 13 (28.9%) | 26 (28.9%) |

***Table S5: Essential Medicines (EMs) Prices Relative to Daily Wages in the Three Included Localities***

|  | | **Locality** | | | | | |
| --- | --- | --- | --- | --- | --- | --- | --- |
|  |  | **Omdurman** | | **Khartoum North** | | **Khartoum** | |
|  |  | **Insured** | **Un-insured** | **Insured** | **Un-insured** | **Insured** | **Un-insured** |
| **Drug equivalence to daily wage with and without insurance coverage in each locality** | **Paracetamol tabs** | 0.15 | 0.51 | 0.15 | 0.61 | 0.14 | 0.54 |
|  | **Mebendazole suspension** | 0.05 | 0.16 | 0.00 | 0.00 | 0.03 | 0.12 |
|  | **Mebendazole tabs** | 0.04 | 0.13 | 0.08 | 0.26 | 0.02 | 0.10 |
|  | **acetyl salicylic acid tabs** | 0.37 | 1.25 | 0.79 | 3.06 | 0.42 | 1.41 |
|  | **Amoxicillin tabs/caps** | 0.21 | 0.85 | 1.00 | 3.87 | 0.47 | 1.82 |
|  | **Amoxicillin suspension** | 0.19 | 0.77 | 0.05 | 0.28 | 0.10 | 0.39 |
|  | **Lidocaine injection** | 0.00 | 0.00 | 0.00 | 0.00 | 0.00 | 0.00 |
|  | **Metronidazole tabs** | 0.18 | 0.72 | 0.15 | 0.72 | 0.20 | 0.80 |
|  | **Artesunate injection** | 0.16 | 0.64 | 0.00 | 0.00 | 0.07 | 0.26 |
|  | **Co-trimoxazole suspension** | 0.09 | 0.34 | 0.04 | 0.14 | 0.02 | 0.10 |
|  | **Artemether Lumefantrine tabs** | 0.00 | 0.00 | 0.00 | 0.00 | 0.12 | 0.50 |
|  | **Ferrous salt tabs** | 0.06 | 0.23 | 0.00 | 0.00 | 0.15 | 0.57 |
|  | **Ferrous salt oral solution** | 0.00 | 0.00 | 0.18 | 0.72 | 0.01 | 0.04 |
|  | **Folic acid tabs** | 0.09 | 0.37 | 0.08 | 0.33 | 0.09 | 0.34 |
|  | **Benzyl benzoate lotion** | 0.00 | 0.00 | 0.00 | 0.00 | 0.00 | 0.00 |
|  | **Fusidic acid ointment** | 0.28 | 1.14 | 0.07 | 0.31 | 0.04 | 0.16 |
|  | **Salbutamol tabs** | 0.09 | 0.35 | 0.01 | 0.05 | 0.04 | 0.13 |
|  | **Ciprofloxacin eye drops** | 0.00 | 0.00 | 0.24 | 0.94 | 0.20 | 0.74 |
|  | **Ringer lactate infusion** | 0.21 | 0.84 | 0.24 | 0.97 | 0.24 | 0.83 |
|  | **Vitamin A caps** | 0.00 | 0.00 | 0.00 | 0.00 | 0.00 | 0.00 |
|  | **Co-trimoxazole suspension for ped** | 0.21 | 0.57 | 0.00 | 0.00 | 0.34 | 0.68 |
|  | **Isoniazid tabs** | 0.38 | 1.51 | 0.00 | 0.00 | 0.00 | 0.64 |

***Table S6: Conservative Conditions in Each PHC’s Outlet Storeroom***

|  | | **Locality** | | | | | | | |
| --- | --- | --- | --- | --- | --- | --- | --- | --- | --- |
|  |  | **Omdurman n=5** | | **Khartoum North n=1** | | **Khartoum n=6** | | **Total n=12** | |
|  |  | **N** | **N %** | **N** | **N %** | **N** | **N %** | **N** | **N %** |
| **Storeroom condition** | **Temperature control method** | 4 | 80.0% | 1 | 100.0% | 6 | 100.0% | 11 | 91.7% |
|  | **Windows/Air vents** | 5 | 100.0% | 1 | 100.0% | 3 | 50.0% | 9 | 75.0% |
|  | **Not exposed to sunlight** | 4 | 80.0% | 1 | 100.0% | 4 | 66.7% | 9 | 75.0% |
|  | **Moisture free** | 5 | 100.0% | 1 | 100.0% | 4 | 66.7% | 10 | 83.3% |
|  | **Cold storage** | 3 | 60.0% | 1 | 100.0% | 1 | 16.7% | 5 | 41.7% |
|  | **Temperature chart** | 0 | 0.0% | 0 | 0.0% | 1 | 16.7% | 1 | 8.3% |
|  | **Medicines not stored in the floor** | 4 | 80.0% | 1 | 100.0% | 2 | 33.3% | 7 | 58.3% |
|  | **Medicines stored systematically** | 5 | 100.0% | 0 | 0.0% | 4 | 66.7% | 9 | 75.0% |
|  | **First expiry first out storage** | 5 | 100.0% | 1 | 100.0% | 4 | 66.7% | 10 | 83.3% |
|  | **No pests evidence** | 5 | 100.0% | 1 | 100.0% | 4 | 66.7% | 10 | 83.3% |
|  | **Tablets not manipulated by hands** | 0 | 0.0% | 0 | 0.0% | 0 | 0.0% | 0 | 0.0% |

***Table S7: Conservative Conditions in Each PHC’s Outlet Storeroom***

|  | | **Locality** | | | | | | | |
| --- | --- | --- | --- | --- | --- | --- | --- | --- | --- |
|  |  | **Omdurman n=7** | | **Khartoum North n=8** | | **Khartoum n=15** | | **Total n=30** | |
|  |  | **N** | **N %** | **N** | **N %** | **N** | **N %** | **N** | **N %** |
| **Dispensary area condition** | **Temperature control method** | 7 | 100.0% | 8 | 100.0% | 15 | 100.0% | 30 | 100.0% |
|  | **Windows/Air vents** | 5 | 71.4% | 8 | 100.0% | 14 | 93.3% | 27 | 90.0% |
|  | **Not exposed to sunlight** | 3 | 42.9% | 5 | 62.5% | 13 | 86.7% | 21 | 70.0% |
|  | **Moisture free** | 4 | 57.1% | 6 | 75.0% | 12 | 80.0% | 22 | 73.3% |
|  | **Cold storage** | 6 | 85.7% | 8 | 100.0% | 14 | 93.3% | 28 | 93.3% |
|  | **Temperature chart** | 2 | 28.6% | 5 | 62.5% | 3 | 20.0% | 10 | 33.3% |
|  | **Medicines not stored in the floor** | 5 | 71.4% | 5 | 62.5% | 9 | 60.0% | 19 | 63.3% |
|  | **Medicines stored systematically** | 7 | 100.0% | 7 | 87.5% | 14 | 93.3% | 28 | 93.3% |
|  | **First expiry first out storage** | 7 | 100.0% | 8 | 100.0% | 12 | 80.0% | 27 | 90.0% |
|  | **No pests evidence** | 6 | 85.7% | 3 | 37.5% | 11 | 73.3% | 20 | 66.7% |
|  | **Tablets not manipulated by hands** | 1 | 14.3% | 5 | 62.5% | 3 | 20.0% | 9 | 30.0% |

References

1. Álvares J, Junior AAG, de Araújo VE, Almeida AM, Dias CZ, Ascef B de O, et al. Access to medicines by patients of the primary health care in the Brazilian Unified Health System. Rev Saude Publica. 2017;51:1s-9s.
