# Supplementary material for: Accessibility crisis of essential medicines at Sudanese primary healthcare facilities: a cross-sectional drugs’ dispensaries assessment and patients’ perspectives
Source: Int J Equity Health. 2023 Oct 17;22:216. doi: 10.1186/s12939-023-02009-y (PMC10583350; doi:10.1186/s12939-023-02009-y)
Supplement: Supplementary file 2 — Supplementary Material 2 [file 12939_2023_2009_MOESM2_ESM.docx]

STROBE Statement—checklist of items that should be included in reports of observational studies

|  | **Item No.** | **Recommendation** | **Page  No.** | **Relevant text from manuscript** |
| --- | --- | --- | --- | --- |
| **Title and abstract** | 1 | (*a*) Indicate the study’s design with a commonly used term in the title or the abstract | 1 | This is a descriptive cross-sectional study |
|  |  | (*b*) Provide in the abstract an informative and balanced summary of what was done and what was found | 1 | **Background**: Access to essential medicines is a critical component of universal health coverage. However, the availability of essential medicines in Sudan isn't well studied. As well, most Sudanese people lack health insurance, making out-of-pocket spending the primary source of drug financing. Therefore, the affordability of medicines in Sudan is questionable, with only 30% of the total population being covered by a public health service or public health insurance. We undertook this study to assess the availability and prices of essential medicines in public-sector health facilities in Khartoum state. Moreover, this study aims at assessing patients' perceived affordability of essential medicines, and accommodation and acceptability of the public facility.  **Methods**: A cross-sectional study was carried-out at 30 primary healthcare facilities' drug dispensaries across three districts in Khartoum state. Within each centre dispensary unit, a standardized checklist evaluated the availability and affordability of 21 essential medicines (EMs) selected from Sudan's national essential medicines list and assessed their storage conditions. Furthermore, at least 13 patients were selected randomly from each dispensary for an exit interview that assessed their perceived accessibility, acceptability, accommodation, and affordability of EMs. Data was collected through the Kobo toolbox and analysed using SPSS version 26.  **Results:** For adult formulae, the overall availability of EMs was determined to be 36.8%, while for paediatric medications, it was 10%. For insured and uninsured individuals, respectively, eight medications and three drugs were judged to be cost-effective. Moreover, the dispensary area conditions were found to be of good quality, yet the storerooms were not functioning in 40% of the outlets. For patients’ variables, only 26.7% had full access. With a low average affordability score (1.5 ± 1.1) from an overall score of 4.  **Conclusion**: Patients had limited access to their needed drugs due to high prices and unfavourable conditions. The health system needs to be strengthened by making essential medicines available and affordable to patients. Ensuring access to free medicines is likely to improve patients’ satisfaction with healthcare services and reduce private expenditure on medicines, which is a long-term, sustainable way towards universal health coverage in Sudan.  **Keywords**: Essential medicines; Primary healthcare services; Affordability; Universal health coverage; Sudan's healthcare. |
| **Introduction** | | | |  |
| Background/rationale | 2 | Explain the scientific background and rationale for the investigation being reported | 2 | The community's perception of the quality of healthcare is most directly measured by the availability of pharmaceuticals, and when essential medications (EM) are exhausted, the load on healthcare facilities decreases by 50–75% (1). The World Health Organization (WHO) defines essential medications as those that address the population's top healthcare requirements. They are chosen with consideration for their significance to public health, proof of their efficacy and safety, and comparative cost-effectiveness (2). A key component of primary healthcare (PHC), according to the Alma-Ata Declaration, is having access to necessary pharmaceuticals (3). Additionally, according to the United Nations (UN), expanding public access to drugs is a step toward achieving universal health coverage and a sign of progress made in the "right to health" movement. (4). Multidisciplinary resources should be mobilized to ensure adequate coverage for essential medicines, as access to pharmaceuticals depends on several economic, political, and social issues and requires joint efforts from the commercial and public sectors (5). Many African countries responded to these recommendations by establishing national policies to control drug availability through the formulation of national essential medicine lists (NEMLs), despite these efforts, a survey conducted by the WHO in the mid-80s revealed that more than 60% of African countries have no access to essential medications (6).  In Sudan, the National Medical Supply Fund (NMSF) indirectly maintains public sector procurement through local and global bids. The commodities (78% of the list of essential medications) are mostly provided to state hospitals and public institutions. Sudan is entirely reliant on imported medications (7), with local manufacture accounting for barely 5% of essential medication output in Sudan (8). There is evidence that restricted local production is the principal barrier to accessing medications in many countries, including Sudan (9) (Alhadi6). Furthermore, studies and reports inspecting the availability of essential medicines in Sudan are scarce and outdated; the only governmental endeavor was the introduction of an ER-RHAD-based health-care program in response to the Bamako Initiative (BI), which was proposed in 1987 at the Annual Meeting of Health Ministers in Bamako. The ER-RHAD program reported that common drugs were available at a rate of 43.9% (10). In furtherance, a national study published in 2009 reported an 85% availability rate for essential medicines in the primary and governmental sectors, but with low-quality storage for these medications (8). As well, the WHO reported in their last assessment of 2018 a 48.6% national availability in Sudan (11). Worldwide, it’s known that many people cannot afford medicines because of their high prices (2). Budgets for pharmaceuticals are high across the board, which is more obvious in developing nations where expenditure on medications makes up a sizeable fraction of overall healthcare costs (4,5,12–14), ranging between 20% and 60% (12), compared to 18% in developed nations (15). Nevertheless, the Sudanese pharmaceutical market is relatively modest, with the total value of the pharmaceutical market (TPM) according to National Medicines and Poisons Board (NMPB) figures projected to be US $650 million, with the public sector accounting for $155 million USD (24.0%) and the private sector accounting for $495 million USD (76.0%) of that total value (7). Moreover, the 1995-founded National Health Insurance Fund (NHIF) is in charge of ensuring that insurance-covered people across the nation have access to basic medical care. Due to the fact that, as of 2014, just 37.3% of people had insurance, out-of-pocket expenses accounted for the majority of the purchases made by the remaining population (16). Although there are no official statistics on access to drugs in Sudan, estimates for household spending on medications in Khartoum state show that they make up 58% of all household healthcare spending. This makes it crucial to check at how affordable pharmaceuticals are in Sudan, particularly given that poverty is pervasive and is thought to be at a rate of more than 50% (17). |
| Objectives | 3 | State specific objectives, including any prespecified hypotheses | 3 | This information prompted us to evaluate the price and availability of vital drugs as well as the quality aspects of each PHC dispensary pharmacy. Furthermore, our research looked into the many drivers and barriers to the usage of public dispensaries, including the accommodation and acceptability of each dispensary. |
| **Methods** | | | |  |
| Study design | 4 | Present key elements of study design early in the paper | 4 | This is an observational, cross-sectional, facility-based study. |
| Setting | 5 | Describe the setting, locations, and relevant dates, including periods of recruitment, exposure, follow-up, and data collection | 4 | conducted in primary healthcare centers in Khartoum state, the capital of Sudan, located in its heart at the confluence of the Blue Nile and White Nile. It contains seven districts. There are a total of 432 primary healthcare facilities, from centers to units and dressing stations, providing preventive and curative health services for insured and uninsured patients. According to WHO Sudan, there are 1.5 primary healthcare facilities for every 10,000 people in Sudan. They are run by physicians and offer packages of services such as childhood immunization, nutrition, reproductive health (RH), integrated management of childhood immunization (IMCI), management of common illnesses, and prescription of necessary drugs (20).  Data was collected between October 11 and October 31, 2022. We followed the STROBE (Strengthening the Reporting of Observational Studies in Epidemiology) guidelines. |
| Participants | 6 | (*a*) *Cohort study*—Give the eligibility criteria, and the sources and methods of selection of participants. Describe methods of follow-up  *Case-control study*—Give the eligibility criteria, and the sources and methods of case ascertainment and control selection. Give the rationale for the choice of cases and controls  *Cross-sectional study*—Give the eligibility criteria, and the sources and methods of selection of participants | N/A |  |
|  |  | (*b*) *Cohort study*—For matched studies, give matching criteria and number of exposed and unexposed  *Case-control study*—For matched studies, give matching criteria and the number of controls per case | N/A  N/A |  |
| Variables | 7 | Clearly define all outcomes, exposures, predictors, potential confounders, and effect modifiers. Give diagnostic criteria, if applicable | 4 | We included all patients aged 18 years and above who were dispensing medicines from the center's outlet dispensary. We included the centers fulfilling the following : (a) governmental primary health care centers run by physicians in Khartoum State; (b) containing outlet pharmacies or medicine dispensaries within them; (c) being active within the past 6 months; and (d) having patients rate of more than 30 patients per day. According to specialists, centers with less than 30 patients per day tend to be irregularly open; only on specific days and hours; and their pharmacies are inoperable.  Patients who took part in the pilot study were omitted, as were those who were seriously unwell because collecting data from them was likely to be impossible. Additionally, all facilities classified as being below primary healthcare facilities in the level of care pyramid were omitted, including PHC units run by community health workers, dressing stations run by nurses, and dispensaries run by medical assistants.  Fifteen key medicines (19 dosage formulae) were selected as per the WHO recommendations in their operational package for pharmaceutical situation assessment (21). To ensure medications align with the acute, chronic, and endemic disease map of Sudan, an advisory group of four experts, including pharmacists, family physicians, and academics, was asked to independently highlight the 15 most needed medications to be included. Their selections were cross-matched by the researchers, and the final 15 medicines selected were listed. Of the 15 medicines, 10 were listed in the global medicine list of the WHO / HAI according to the disease spectrum and necessity for basic medical care worldwide (19). All of the medicines were listed in Sudan's last (2019) NEML (22). |
| Data sources/ measurement | 8* | For each variable of interest, give sources of data and details of methods of assessment (measurement). Describe comparability of assessment methods if there is more than one group | N/A |  |
| Bias | 9 | Describe any efforts to address potential sources of bias | 6 | For the patients’ data, a structured and pre-tested questionnaire was adopted from the Brazilian PAUMA study "National Survey on Access, Use, and Promotion of Rational Use of Medicines "(18), which was also dependent on Penshansky and Thomas' concept. To verify the precision and reliability of the PAUMA instrument we used in this study, and to ensure its cultural suitability, an expert panel assessed and confirmed the instrument's content. The questionnaire was then translated to Arabic by the study author and translated back to English by language experts; the two copies were compared for reliability. Pilot research was then carried out among a group of 50 patients from different primary healthcare centers. The questionnaire was then edited by the authors accordingly. |
| Study size | 10 | Explain how the study size was arrived at | 5 | The estimated sample size of health centers was chosen with a 95% confidence interval and 15% margin of error (e), from 91 total eligible centers (N). The target sample size was therefore 30 centers derived from the following simple formula (23):  $n=\frac{N}{1+{N(e)}^{2}}$ = 30  For the patients sample the following formula was used$, n =\frac{z^{2}P\left( 1-P \right)}{e^{2}}$ with a 95% confidence interval (CI), 50% response distribution, and 5% margin of error; a sample of 384 was considered as the minimal sampling to represent the study population. by multiplying it by 1.2 design effect and considering a 35% non-response rate. the final sample size was 630.  Multi-stage cluster sampling was used to select the sample facilities and patients. Stage one was the random selection of three representative localities from the seven localities of Khartoum state. Omdurman, Khartoum North (Bahri), and Khartoum were selected using simple random sampling. Stage two was the selection of the PHC centers; using probability proportionate to size, 15 centers from Khartoum, seven from Omdurman, and eight from Bahri were randomly selected. Stage three was the selection of patients, due to the approximate equal patient rate at each center, the sample size was divided relatively equally between the 30 centers. Using the relative patient rate at each outlet pharmacy, an interval was created by each data collector, and a systematic random sampling method was used. Ultimately, at least 13 responses were collected from each dispensary unit. |

Continued on next page

| Quantitative variables | 11 | Explain how quantitative variables were handled in the analyses. If applicable, describe which groupings were chosen and why | 6 | . ***Patients’ data*** was downloaded from KoboCollect into an Excel sheet file and then cleaned manually. Analysis was performed using SPSS version 26 (Statistical Package for Social Sciences). All missed variables were coded. |
| --- | --- | --- | --- | --- |
| Statistical methods | 12 | (*a*) Describe all statistical methods, including those used to control for confounding | 6 | Descriptive statistics were used for the patients’ characteristics, the availability of drugs, and the mean of the prices; the outcomes were displayed in tables and figures. Analysis of variance (ANOVA), independent sample t-test, and spearman’s rho correlation tests were used to find the association between the different study variables |
|  |  | (*b*) Describe any methods used to examine subgroups and interactions | N/A |  |
|  |  | (*c*) Explain how missing data were addressed | - |  |
|  |  | (*d*) *Cohort study*—If applicable, explain how loss to follow-up was addressed  *Case-control study*—If applicable, explain how matching of cases and controls was addressed  *Cross-sectional study*—If applicable, describe analytical methods taking account of sampling strategy |  |  |
|  |  | (*e*) Describe any sensitivity analyses | N/A |  |
| **Results** | | | | |
| Participants | 13* | (a) Report numbers of individuals at each stage of study—eg numbers potentially eligible, examined for eligibility, confirmed eligible, included in the study, completing follow-up, and analysed | 6 | ***Availability Index:*** Availability of essential drugs refers to, by the WHO/HAI, the proportion of the surveyed institutions that can provide a certain drug to the total number of survey institutions (19). The mean availability of the selected medicines was calculated per the WHO/HAI recommendations. Furthermore, we calculated the availability of each medicine as the percent availability of the total assessed medicines at the surveyed PHC facility and compared the availability for the different study districts.  ***Affordability Index:*** Is defined by the WHO/HAI, as the affordability of an essential medication during a specific course of treatment, the total medicine cost for the treatment of a condition with standard dosages of medicines divided by the minimum daily wage for non-technical staff in government departments (19). Each essential drug price for the complete treatment course was collected in Sudanese SDG, then divided by 311 SDGs, which is the daily lowest-paid unskilled government worker wage for Sudan as extracted from the World Salaries website (24). If the total expense of drug treatment is less than the aforementioned minimum daily income criterion, the drug is assumed to be more affordable, and vice versa. For price evaluation, the ***median price ratio (MPR***), which indicated the ratio of one medicine's unit price to the international reference price (IRP) (19), was utilized. MPR's particular calculating formula is as follows: MPR = median unit price of the target drug within the survey range/international reference price × 100%. When comparing drug purchase price levels using MPR values, MPR = 1 is typically used as the threshold value. When this value is less than one, it means that the investigated drug price is lower than the international average standard, and vice versa. The WHO recommends that retail pricing of medications should not allow for an MPR in excess of 2.(25). |
|  |  | (b) Give reasons for non-participation at each stage | N/A |  |
|  |  | (c) Consider use of a flow diagram | N/A |  |
| Descriptive data | 14* | (a) Give characteristics of study participants (eg demographic, clinical, social) and information on exposures and potential confounders | 7 | The study assessed services at 30 primary healthcare centers distributed in Khartoum (52.5%), Khartoum North (28.1%), and Omdurman (19.4%) localities***.*** The total number of respondents was 630, with a mean age of 43.7 ± 15 years, with more than two-thirds (69.7%) being females. Nearly 60% were unemployed, while 25.1% reported having no access to health insurance. 67.5% of the participants reported the purpose of their visit to be the attainment of a drug for a current (acute) illness |
|  |  | (b) Indicate number of participants with missing data for each variable of interest | 8 | Table 1 |
|  |  | (c) *Cohort study*—Summarise follow-up time (eg, average and total amount) | N/A |  |
| Outcome data | 15* | *Cohort study*—Report numbers of outcome events or summary measures over time | N/A |  |
|  |  | *Case-control study—*Report numbers in each exposure category, or summary measures of exposure | N/A |  |
|  |  | *Cross-sectional study—*Report numbers of outcome events or summary measures | 9,10,11,12,13,14,15 |  |
| Main results | 16 | (*a*) Give unadjusted estimates and, if applicable, confounder-adjusted estimates and their precision (eg, 95% confidence interval). Make clear which confounders were adjusted for and why they were included | 3 | Considering a confidence level of 95%, |
|  |  | (*b*) Report category boundaries when continuous variables were categorized | N/A |  |
|  |  | (*c*) If relevant, consider translating estimates of relative risk into absolute risk for a meaningful time period | N/A |  |

Continued on next page

| Other analyses | 17 | Report other analyses done—eg analyses of subgroups and interactions, and sensitivity analyses | 5,6,7 |  |
| --- | --- | --- | --- | --- |
| **Discussion** | | | | |
| Key results | 18 | Summarise key results with reference to study objectives | 16 | This study has evaluated patients' accessibility to essential medicines at the primary healthcare level in Khartoum, Sudan, through a full analysis of the availability, acceptability, accommodation, and affordability. And to make relevant suggestions to improve the current situation. We have attempted to reflect the exact situation of availability and prices of 19 EMs in 30 outlet dispensaries of Sudan PHCs. To the best of our knowledge, this is the first study that evaluates accessibility by both examining patients’ perspectives and analysing pharmacy circumstances in Sudan and maybe in the African region. |
| Limitations | 19 | Discuss limitations of the study, taking into account sources of potential bias or imprecision. Discuss both direction and magnitude of any potential bias | 18 | Finally, this study was limited to the public dispensaries of Khartoum state, so more comprehensive research is needed to conclude the national situation. The WHO/HAI methodology is based on on-shelf availability; therefore, this study might not indicate stock availability; however, this methodology is widely used in the literature, and it puts our findings in a global context |
| Interpretation | 20 | Give a cautious overall interpretation of results considering objectives, limitations, multiplicity of analyses, results from similar studies, and other relevant evidence | 19 | Conclusion |
| Generalisability | 21 | Discuss the generalisability (external validity) of the study results |  |  |
| **Other information** | |  | | |
| Funding | 22 | Give the source of funding and the role of the funders for the present study and, if applicable, for the original study on which the present article is based | 20 | No funding was received for conducting this study. |

*Give information separately for cases and controls in case-control studies and, if applicable, for exposed and unexposed groups in cohort and cross-sectional studies.

**Note:** An Explanation and Elaboration article discusses each checklist item and gives methodological background and published examples of transparent reporting. The STROBE checklist is best used in conjunction with this article (freely available on the Web sites of PLoS Medicine at http://www.plosmedicine.org/, Annals of Internal Medicine at http://www.annals.org/, and Epidemiology at http://www.epidem.com/). Information on the STROBE Initiative is available at www.strobe-statement.org.
