# Supplementary material for: Accessibility crisis of essential medicines at Sudanese primary healthcare facilities: a cross-sectional drugs’ dispensaries assessment and patients’ perspectives
Source: Int J Equity Health. 2023 Oct 17;22:216. doi: 10.1186/s12939-023-02009-y (PMC10583350; doi:10.1186/s12939-023-02009-y)
Supplement: Supplementary file 3 — Supplementary Material 3 [file 12939_2023_2009_MOESM3_ESM.docx]

**Demographic**

1. *Name of the center and locality*: ()

2. *Gender*: (1) Male. (2) Female.

3. *Age*: (_____) years.

4. *Marital status:*

(1) Single

(2) Married

(3) Divorced

(4) Widowed

(5) Others

5. *Educational level:*

(1) Illiterate

(2) Informal education (Khalwa)

(3) Primary school

(4) Secondary school

(5) Bachelor/university degree

(6) Higher education degree

7. *Occupational status*

(1) Occupied

(2) Not occupied

8. *How much does your household spend monthly on regular expenses in SDGs?*

(Regular expenses= food, transport, clothing, school fees, water and Health Services)

**Overall access:**

1. *What is the purpose of your dispensary visit?*

(1) Dispensing regular medications

(2) Dispensing a currently prescribed medicines

(3) Over the counter medications

2. *How do you perceive your access to your needed medications?*

(1) Full access

(2) Partial access

(3) No access

**Accessibility**

*1. Is the primary healthcare center far from the patients' house?*

(1) Yes

(2) More or less

(3) No

2. *What is your mean of transport to reach the PHC center?*

(1) General

(2) Walking

(3) Private car/motorbike

(4) Others

3. *Are there any existing signs in the PHC center to find the medicines dispensing unit?**

(1) Yes

(2) No

**Accommodation**

1. *Do you regard this dispensing unit as comfortable?*

(1) Yes

(2) More or less

(3) No

2. *Do you regard this dispensing unit as clean?*

(1) Yes

(2) More or less

(3) No

3. *Please estimate the waiting time in minutes; from reaching the dispensing unit till receiving your medicines?*

In minutes ()

4. *Do you perceive this time as longtime?*

(1) Yes

(2) More or less

(3) No

5. *Are the opening hours of this dispensing unit suitable?*

(1) Yes

(2) More or less

(3) No

**Acceptability**

1. *Do the staff of the dispensing unit treat patients with respect and courtesy?*

(1) Yes, always

(2) Yes, sometimes

(3) No, never

2. *Do you regard the service presented at the PHC unit of good quality?*

(1) Yes

(2) More or less

(3) No

3. *Is your privacy respected throughout the services*?

(1) Yes, always

(2) Yes, sometimes

(3) No, never

**Affordability**

1. *Were you ever been not able to buy something important to cover expenses for any health problem?*

(1) Yes

(2) No

2. *Do you regard the medicines you buy as expensive*?

(1) Yes

(2) More or less

(3) No

(4) I got it for free

*3. Do you have health insurance?*

(1) Yes

(2) No

**Any comments? ()**
